# Interdisciplinary and cross-sectoral perioperative care model in cardiac surgery: implementation in the setting of minimally invasive heart valve surgery (INCREASE)—study protocol for a randomized controlled trial

**DOI:** 10.1186/s13063-022-06455-x

**Published:** 2022-06-23

**Authors:** Susanne G. R. Klotz, Gesche Ketels, Christian A. Behrendt, Hans-Helmut König, Sebastian Kohlmann, Bernd Löwe, Johannes Petersen, Sina Stock, Eik Vettorazzi, Antonia Zapf, Inke Zastrow, Christian Zöllner, Hermann Reichenspurner, Evaldas Girdauskas

**Affiliations:** 1 Department of Physiotherapy, University Medical Center Hamburg-Eppendorf, Martinistr. 52, 20246 Hamburg, Germany; 2 Research Group GermanVasc, Department of Vascular Medicine, University Heart and Vascular Center, University Medical Center Hamburg-Eppendorf, Martinistr. 52, 20246 Hamburg, Germany; 3 Department of Health Economics and Health Services Research, University Medical Center Hamburg-Eppendorf, Martinistr. 52, 20246 Hamburg, Germany; 4 Department of Psychosomatic Medicine and Psychotherapy, University Medical Center Hamburg-Eppendorf, Martinistr. 52, 20246 Hamburg, Germany; 5 Department of Cardiovascular Surgery, University Heart and Vascular Center, University Medical Center Hamburg-Eppendorf, Martinistr. 52, 20246 Hamburg, Germany; 6 Department of Cardiothoracic Surgery, University Hospital Augsburg, Stenglinstr. 2, 86156 Augsburg, Germany; 7 Department of Medical Biometry and Epidemiology, University Medical Center Hamburg-Eppendorf, Martinistr. 52, 20246 Hamburg, Germany; 8 Department of Patient and Care Management, University Medical Center Hamburg-Eppendorf, Martinistr. 52, 20246 Hamburg, Germany; 9 Department of Anesthesiology, Center of Anesthesiology and Intensive Care Medicine, University Medical Center Hamburg-Eppendorf, Martinistr. 52, 20246 Hamburg, Germany

**Keywords:** Minimally invasive heart valve surgery, Enhanced recovery after surgery, ERAS, Fast track, Interdisciplinary, Physiotherapy, Psychosomatic medicine, Advanced practice nurse

## Abstract

**Background:**

Valvular heart diseases are frequent and increasing in prevalence. Minimally invasive heart valve surgery embedded in an interdisciplinary enhanced recovery after surgery (ERAS) program may have potential benefits with regard to reduced length of stay and improved patient reported outcomes. However, no prospective randomized data exist regarding the superiority of ERAS program for the patients’ outcome.

**Methods:**

We aim to randomize (1:1) a total of 186 eligible patients with minimally invasive heart valve surgery to an ERAS program vs. standard treatment at two centers including the University Medical Center Hamburg-Eppendorf, Germany, and the University Hospital Augsburg, Germany. The intervention is composed out of pre-, peri-, and postoperative components. The preoperative protocol aims at better preparation for the operation with regard to physical activity, nutrition, and psychological preparedness. Intraoperative anesthesiologic and surgical management are trimmed to enable an early extubation. Patients will be transferred to a specialized postoperative anesthesia care unit, where first mobilization occurs 3 h after surgery. Transfer to low care ward will be at the next day and discharge at the fifth day. Participants in the control group will receive treatment as usual. Primary endpoints include functional discharge at discharge and duration of in-hospital care during the first 12 months after index surgery. Secondary outcomes include health-related quality of life, health literacy, and level of physical activity.

**Discussion:**

This is the first randomized controlled trial evaluating the effectiveness of an ERAS process after minimally invasive heart valve surgery. Interprofessional approach is the key factor of the ERAS process and includes in particular surgical, anesthesiological, physiotherapeutic, advanced nursing, and psychosocial components. A clinical implication guideline will be developed facilitating the adoption of ERAS model in other heart teams.

**Trial registration:**

The study has been registered in ClinicalTrials.gov (NCT04977362 assigned July 27, 2021).

## Background

Valvular heart diseases are the most common structural heart diseases in adults associated with high morbidity and mortality [1]. Worldwide, the prevalence of valvular heart diseases is increasing, which was related to an increase in morbidity and mortality rates [2]. In 2020, approximately 35,500 isolated heart valve procedures were performed in Germany. While about 55% of the mitral valve operations were executed using minimally invasive approaches, the number of minimally invasive aortic valve procedures constituted about 37% [3].

Minimally invasive valve surgery was not only associated with less pain, better cosmetic result, faster return to preoperative function, and reduced length of stay (LOS) [4], but also with higher level of physical activity [5]. Higher level of in-hospital physical activity was inversely correlated with post-operative LOS [6]. The amount of physical activity in patients undergoing minimally invasive surgery may be increased additionally with the aid of daily individualized physiotherapy [7], emphasizing the need of physiotherapy in the care process.

The extension of interprofessional care process by targeted preoperative psychosomatic intervention may have additional benefits for the patients in terms of shorter LOS [8]. Moreover, patients could be motivated to be active partners in the whole treatment process, be more physically active, and gain more perceived self-control [9].

A contemporary approach to not only integrate the different professions, but also to cumulatively improve the recovery of patients from surgical procedures is an Enhanced Recovery After Surgery (ERAS) protocol. ERAS aims at reducing physical and psychological stress of surgery and improving outcomes with the help of a coordinated preoperative, perioperative, and postoperative management process [10]. Although initially introduced in the colorectal and orthopedic surgery, ERAS programs are also being increasingly adopted by cardiac surgery. Compared to the standard care, ERAS results in lower rates of postoperative atrial fibrillation, earlier extubation, shorter intensive care length of stay, and shorter overall length of hospital stay [11, 12]. In addition, ERAS may decrease pain intensity, reduce opioid use, increase early postoperative mobility, accelerate oral diet, and decrease costs in valve surgery [11, 12]. However, previous studies comprise almost exclusively full sternotomy procedures, primarily coronary artery bypass grafting [12]. Of note, the evidence for ERAS in the minimally invasive heart valve procedures is limited to a single observational study showing lower pain levels and reduced LOS in the ERAS-treated patient cohort [13].

### Objectives

Prior to this study, a modern fast-track protocol for minimally invasive heart valve procedures was developed at the University Medical Centre Hamburg-Eppendorf. Preliminary analyses of the pilot phase directly after ERAS implementation showed good clinical feasibility of the ERAS protocol with high adherence of the patients to it. Moreover, an appropriate safety profile has been demonstrated, resulting in no complication directly attributable to the ERAS protocol [14]. Furthermore, the intensive care unit LOS and the total LOS were significantly reduced and the total costs for the in-hospital stay per patient were significantly lower as compared to those patients undergoing minimally invasive heart valve surgery according to previous hospital standards [15].

The aim of our current study is to investigate the effectiveness of this further developed and refined interprofessional ERAS protocol in patients undergoing minimally invasive heart valve surgery in comparison to the standard treatment as usual.

## Methods/design

### Study design

This study is designed as a randomized controlled trial with two study arms taking place in an academic tertiary care setting in Germany. The intervention group will receive treatment according to the ERAS protocol and the control group will undergo the standard treatment as usual. An interdisciplinary and cross-sectional consortium consisting of two university medical centers, seven referring hospitals, and eight rehabilitation hospitals is responsible for the INCREASE project. In addition, the health insurance company “BARMER” and the patient organization “German Heart Foundation” are included in the project as active members of the consortium.

The study protocol (version 10; May 17, 2021) is in accordance with the SPIRIT 2013 statement [16], and all procedures in the trial will be conducted in accordance with the Helsinki Declaration [17]. Protocol modifications will be communicated to relevant parties.

### Sample and recruitment

Study subjects will be equally recruited at the University Medical Center Hamburg-Eppendorf and at the University Hospital Augsburg, both Germany. All patients scheduled for minimally invasive heart valve surgery (i.e., mitral valve or aortic valve surgery) as primary inclusion criteria will be contacted by screening call and the study objectives will be introduced. If patients are keen to participate and give their formal consent, a final eligibility screening is conducted. During this final screening assessment, the study-specific inclusion and exclusion criteria will be verified. Inclusion criteria are as follows:

- Indication for elective minimally invasive aortic or mitral valve surgery
- Ability to adequately understand the nature and extent of the individual's requirements for participation in the ERAS model of care
- Written informed consent
- Functional status classification as “FIT” or “Pre-FRAIL” with the LUCAS functional index derived from the Longitudinal Urban Cohort Ageing Study (LUCAS) [18]

Patients will be excluded if they had one of the following conditions or co-morbidities at baseline:

- Severe chronic obstructive pulmonary disease (GOLD III or IV)
- Dialysis-dependent renal failure
- Advanced liver cirrhosis (Child stages B + C)
- Residual neurological impairment after prior stroke, especially leg-related hemiplegia, major restrictions of mobility, and/or neuropsychological disorders
- Predicted life expectancy < 1 year (e.g., advanced malignancy)
- Previous cardiac surgery (i.e., relative contraindication for minimally invasive technique)
- Severe depressive disorder
- Substance-related addictive disorders (e.g., alcohol, drugs)
- Lack of a social environment that ensures an adequate supportive care during perioperative course

If patients are eligible to participate, they will receive in-detail study information via telephone and by post. Potential participants will have the opportunity to ask questions directly during the telephone call or at any time later on via email, telephone, or personal contact depending on their personal preference. After the screened candidates confirm their willingness to participate, they will sign the written informed consent.

### Sample size calculation

Sample size calculation is based on the two co-primary endpoints: (a) total days in hospital due to cardiac causes during the first postoperative year and (b) physical performance measured by the Six-Minute Walk Test (6MWT). For the first primary co-endpoint, a superiority hypothesis was established expecting the hospitalization time in the intervention group to be shorter than in the control group. Regarding the second primary co-endpoint, a non-inferiority hypothesis is assumed stating that participants in the intervention group, although being discharged earlier, will achieve comparable values to the control group in the physical performance measurement. For the study success, both primary null hypotheses must be rejected.

Because these are co-primary endpoints, adjustment of the type I error for multiple testing is not required. Type I error is fixed at *α* = 0.025 based on the one-sided test. However, because the power is affected by the chosen study design, it is set to 90% for the single hypotheses to ensure an overall power of at least 80%.

In terms of superiority hypothesis, no precise data are available regarding in-hospital days for cardiac reasons during the first year after surgery. Nonetheless, we assume that the total number of in-hospital days does not differ between both groups after initial discharge after surgery. Therefore, the mean primary in-hospital stay served as a background for sample size calculation. For this purpose, we used the data derived from internal evaluation of the University Medical Centre Hamburg-Eppendorf during the pilot phase of ERAS program. The mean LOS was 6.1 days with a standard deviation (SD) of 2.5 days in the intervention group, as compared to 8.0 days with an SD of 4.1 days in the control group. Using the two-sample *t* test based on unequal variances, the sample size needed to demonstrate the superiority in the intervention group was 69 patients per group, or 138 patients in total.

Regarding the non-inferiority hypothesis on the physical performance between the study groups, a mean walking distance of 300 meters with an SD of 89 meters is assumed for the standard surgical procedure [19]. These results are also expected for the intervention group. A deviation > 15 percentage (i.e., > 45 m) was defined as relevant (non-inferiority boundary), based on clinical considerations. Using the two-sample *t*-test based on equal variances, the number of cases to prove the non-inferiority was 84 patients per group, or 168 patients in total.

Thus, to successfully complete the study, a total of 168 patients (maximum of the two case numbers) are required. To compensate for possible dropouts, additional 10% (*n* = 16.8 ≈ 18) participants were calculated. This final adjustment results in a total subject number of *N* = 186 (*n* = 93 per treatment arm).

### Randomization and blinding

Patients will be randomized (1:1) after written informed consent and study inclusion prior to any treatment arm-specific intervention. Central block randomization with variable block length will be used. Randomization will be stratified by the institution of treatment. Since no prognostically relevant variables are known in the included patient population, no further variables will be stratified for at randomization.

The randomization codes will be generated by a biostatistician using a random number generator. A third person not involved in the randomization process will prepare opaque sealed envelopes containing the participant’s order on the outside and the participant’s group allocation on the inside. Study nurses in both study centers will open the envelopes in the designated order to allocate the participants. Thus, the nondisclosure of the group allocation will be ensured.

Blinding of the participants and health care professionals will not be possible due to the different interventions in both study groups. Blinding of the assessors of the performance measurements will also not be possible due to the different time points of measurements; however, the assessors will not be involved in the treatment process in the two groups. The statistical analysis of the study data will be performed by blinded statisticians.

### Intervention group

The intervention group will receive a multimodal interprofessional intervention targeted at faster postoperative recovery, active participation of the patient in the treatment process, optimization of the clinical outcome, and improved quality of life. This specialized approach, the INCREASE program, is the further development of the already piloted fast track protocol for minimally invasive heart valve surgery at the University Medical Centre Hamburg-Eppendorf [14, 15] and is in accordance with ERAS guidelines in cardiac surgery [20]. The key features of the intervention can be divided into preoperative, intraoperative, and postoperative components. Although the key features are determined in advance, deviations might occur due to the individual needs of the different patients.

One unique characteristic of the program is the INCREASE nurse, an advanced practice nurse who is the primary contact person for the patients and their relatives throughout the whole perioperative process. The nurse supports closely the patients before, during, and after hospital stay, whereas the contact before and after the hospital stay is primarily organized via telephone or e-mail. During the in-hospital stay, the nurse is closely present on the general ward and on the specialized postoperative anesthesia care unit (PACU).

Another unique characteristic of the INCREASE program is the use of motivational interviewing [21] strategies in the communication with the patient. Using motivational interviewing increases intrinsic motivation of the patients and helps to strengthen their active role in the treatment process. In addition, motivational interviewing enables patients to set their own goals guiding and structuring the process with these goals. The patients will set their own goals for the discharge (short term), after 3 months postoperatively (intermediate), and after 12 months post-surgery (long term). Furthermore, daily goals during in-hospital stay will be determined by the patients, which serve as motivator and could increase adherence to the INCREASE program.

### Preoperative components

During the time interval of 2 to 4 weeks prior to scheduled surgery, patients and additional person of their choice will attend an outpatient educational session. This interprofessional meeting will last approximately 3 h and will consist of structured baseline interview with the surgeon, the anesthetist, the INCREASE nurse, the physiotherapist, and the psychologist.

One crucial component of the intervention during the educational session is the introduction of a specifically designed INCREASE diary for the participants. This diary includes relevant information about their in-hospital stay but also contains several worksheets for the participants. It is designed as a patient-oriented guide throughout the preoperative phase to support the nutrition, physical activity, and psychosocial preparation. Furthermore, it serves as an instrument to monitor their physical activity level, pain management, and nutrition during the in-hospital stay. Additionally, the diary comprises cognitive exercises for the period between the education session and the operation to support a cognitive-affective level, to develop individual coping strategies, and to become an active part in the treatment process.

During the educational meeting, the surgeon and the anesthetist focus on the surgical procedure as well as the perioperative anesthesiologic management. The INCREASE nurse give an overview of the whole in-hospital stay and the routine medical and nursing care procedures. Further issues are the nutrition including preoperative carbohydrate loading for the last 10 days preoperatively and management of postoperative pain as well as the prophylaxis of postoperative nausea and vomiting (PONV). Moreover, the INCREASE nurse introduces and explains the patient diary and carries out a nursing anamnesis with the patient. The physiotherapist advises the patients regarding physical activity including its effects on the bio-psycho-social health and discusses an individualized pre-habilitation program. The crucial role of physical activity during in-hospital stay will be highlighted as well as the empowerment of patients through dressing in their daily clothes instead of nightwear throughout the day. The psychologist aids the patients to develop their strategies to acquire an active role in the treatment process and to handle situations associated with cognitive-affective stress like pain or other symptoms. In order to facilitate a transition as seamless as possible from the inpatient setting to rehabilitation, follow-up treatment is already organized together with the patient during this consultation.

Patients will be admitted to hospital on the day prior to surgery. The INCREASE nurse, the physiotherapist, and, if necessary or requested from the patient, the psychologist visit the patient on the day of the hospital admission. During this consultation, the experiences of the pre-habilitation period are reflected and the questions raised are answered. Moreover, the patients are encouraged to stay physically active during the whole in-hospital stay and to dress up. Prior to surgery, the patients receive a carbohydrate drink as bolus to reduce the insulin resistance and to improve glucose control. In addition, oral premedication with midazolam is administered.

### Intraoperative components

Anesthesiologic and surgical management in the ERAS program has been described in detail previously [14]. Briefly, cardiopulmonary bypass flow is targeted to > 3.2 l/m^2^/min and core temperature is lowered to 32–34 °C. Restrictive fluid therapy is implemented during CPB with the goal of negative fluid balance at the end of the procedure. The patient is rewarmed to 37.0 °C by means of the cardiopulmonary circuit and a heating blanket.

The minimally invasive access is used in all patients undergoing valvular surgery. While mitral valve surgery with or without concomitant tricuspid valve surgery, left atrial ablation, and closure of left atrial appendage is addressed through a right anterolateral incision in the fourth intercostal space, aortic valve surgery is routinely performed via a partial upper J mini sternotomy in the third intercostal space. 3D full-endoscopic non-rib spreading approach with a soft-tissue retractor is implemented in mitral valve surgery. Both surgical approaches aim to maintain the stability of the chest and, therefore, to enable early ambulation.

### Postoperative components

All patients are weaned of mechanical ventilation and extubated in the operating room before transferring them to PACU. In the PACU, patients are supported by intermittent non-invasive mechanical ventilation. Pain therapy and PONV prophylaxis are carried out according to the standardized protocol, as described previously [14]. First physiotherapy with a focus on early mobilization is carried out 3 h after surgery. Patients have their first postoperative food intake in the evening in the sitting position. In case of an uneventful postoperative course chest tubes, invasive arterial line, and central venous catheter are removed during the first 12 h postoperatively and before transfer to the low care ward early in the next morning.

At the low care ward, PONV prophylaxis and pain therapy are continued as needed. An interprofessional round consisting of INCREASE nurse, surgeon, anesthetist, physiotherapist, and psychotherapist visit the patients every morning and discuss the recovery process and any potential events that occurred during the past day. In addition to the ward rounds, the INCREASE nurse carries out further nursing visits to patients in order to ensure extended nursing care and to detect possible complications at an early stage. Physiotherapy is continued to guide the patients while increasing their physical activity and taking responsibility of their own recovery. The interventions are tailored to meet the individual discharge goal of patients. The psychotherapist supports the patients on an individualized base, as required. Pacemaker wires and peripheral venous line are routinely removed on the third postoperative day. Discharge, either to rehabilitation or to home environment, takes place approximately on the fifth postoperative day.

### Control group

Having an indication for elective minimally invasive aortic or mitral valve surgery, all participants need to be treated; hence, an active comparator as control group was chosen. The control group receive the treatment as usual (TAU) according to the established standards in the two study sites and depending on individual needs. In contrast to the intervention group, they will not receive an outpatient educational session prior to admission and will be transferred intubated to the intensive care unit (ICU) instead of being transferred to the specialized PACU. These patients receive only routine physiotherapy according to the standard of care that is one visit at the intensive care unit and one visit at normal ward, unless individual indications are present. Furthermore, no psychosomatic support and counseling by an INCREASE nurse will be included.

### Time points of measurements

**Fig. 1:**
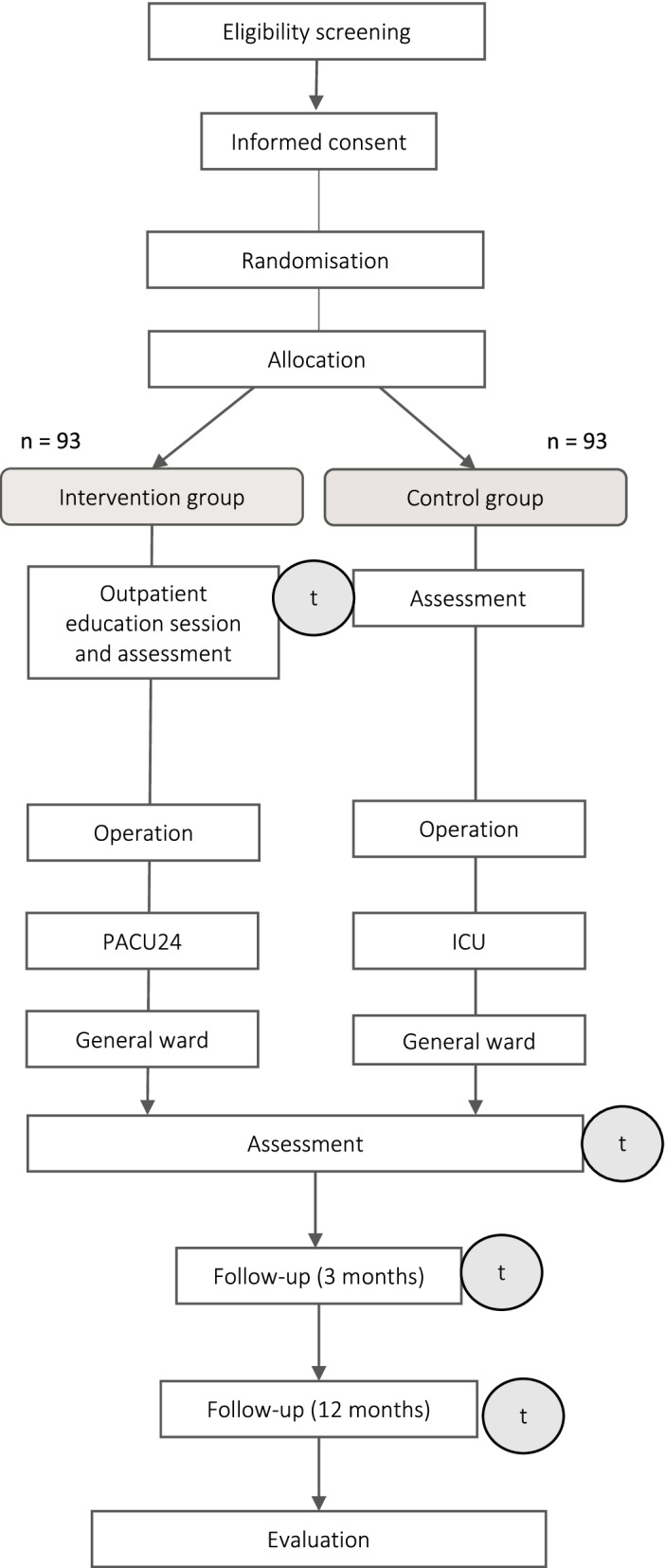
Overview of study procedure

Measurements will be taken at baseline (t0), at the end of the in-hospital stay (t1), after a follow-up period of 3 months (t2), and after a follow-up period of 12 months (t3). Baseline measurement will be conducted before any group-specific intervention occurs. Therefore, the baseline assessment (t0) in the intervention group will be conducted right before the outpatient educational session, whereas in the control group, it will be on the first day of in-hospital stay, which is routinely the day before the surgery. The t1 assessment will be conducted at the last day before discharge. Moreover, further clinical data that depict the treatment process during the in-hospital stay will be collected. Figure 1 summarizes the study flow diagram and the time points of measurements.

### Baseline characteristics and in-hospital data

The following data will be collected preoperatively: main diagnosis and secondary diagnoses, sex, age, marital status, education, and professional qualification. During the operative procedure, medication-related variables (e.g., analgesics, muscle relaxants, antiarrhythmics, and PONV), transfusions, infusions, diuresis, lactate values, and hemoglobin values as well as values from the electrocardiogram and the cardiopulmonary bypass will be gathered. Furthermore, all clinically relevant events, the results of diagnostic procedures, medication (e.g., analgesics, antiemetics), the intensity of pain, PONV, dizziness, or delirium, as well as nursing procedures and physiotherapeutic/psychosomatic interventions during the in-hospital stay will be recorded. In addition, adverse events and serious adverse events respectively as well as all dropouts during the whole study period will be captured.

### Outcomes

#### Primary outcomes

Two indicators are set as co-primary endpoints, while both a priori null hypotheses must be rejected for reaching the study success:

1. Total days in hospital due to cardiac causes (including the initial in-hospital stay for surgery) during the first postoperative year
2. Functional performance measured by the 6MWT [22] at the time point of hospital discharge

#### Secondary outcomes

Several secondary outcome measures will be used. See Table 1 for an overview of the different measurement time points for specific assessments.

- Goal Attainment Scale (GAS) [23] for determination and assessment of individualized participants’ goals in the intervention group at discharge (short-term goal), after 3 months (intermediate goal), and after 12 months (long-term goal)
- German version [24] of the HeartQoL [25] measuring health-related quality of life, especially in patients with heart disease
- German version [26] of the 5-level EQ-5D health-related quality of life questionnaire (EQ-5D-5L) [27]
- German version [28] of the Brief Illness Perception Questionnaire (BIPQ) [29] for assessment of individualized illness conceptions
- Somatic Symptom Scale—8 (SSS-8) [30] for the measurement of somatic symptom burden
- Somatic Symptom Disorder—B Criteria Scale (SSD-12) [31] to evaluate the dealing with somatic symptoms
- German version [32] of the Cardiac Anxiety Questionnaire (CAQ) [33] for quantitative assessment of cardiac anxiety
- Patient Health Questionnaire-9 (PHQ-9) [34] for the assessment of depression severity
- Generalized Anxiety Disorder 2-item version (GAD-2) [35] for the measurement of anxiety severity
- German version [36] of the Life Orientation Test—revised (LOT-R) [37] to assess the dispositional optimism
- German version [38] of the International Physical Activity Questionnaire Short Form (IPAQ-SF) [39] to evaluate the level of physical activity
- 6MWT to quantify the functional performance at 12 months postoperatively
- Timed Up and Go (TUG) [40] to capture the functional performance
- One Minute Sit to Stand Test (1STS) [41] for the evaluation of physical performance
- Dynamometry to rate the hand grip [42]
- European Health Literacy Questionnaire HLS-EU-Q16 [43] to measure health literacy
- Treatment Expectation Questionnaire (TEX-Q) [44] to assess treatment expectations
- German version [45] of the short form of the Readiness for Hospital Discharge Scale (RHDS) [46] for the assessment of readiness for hospital discharge
- Questionnaire for Health-Related Resource Use in an Elderly Population [Fragebogen zur Inanspruchname medizinischer und nicht-medizinischer Versorgungsleistungen im Alter] (FIMA) [47] to evaluate the use of health care services

**Table 1:**
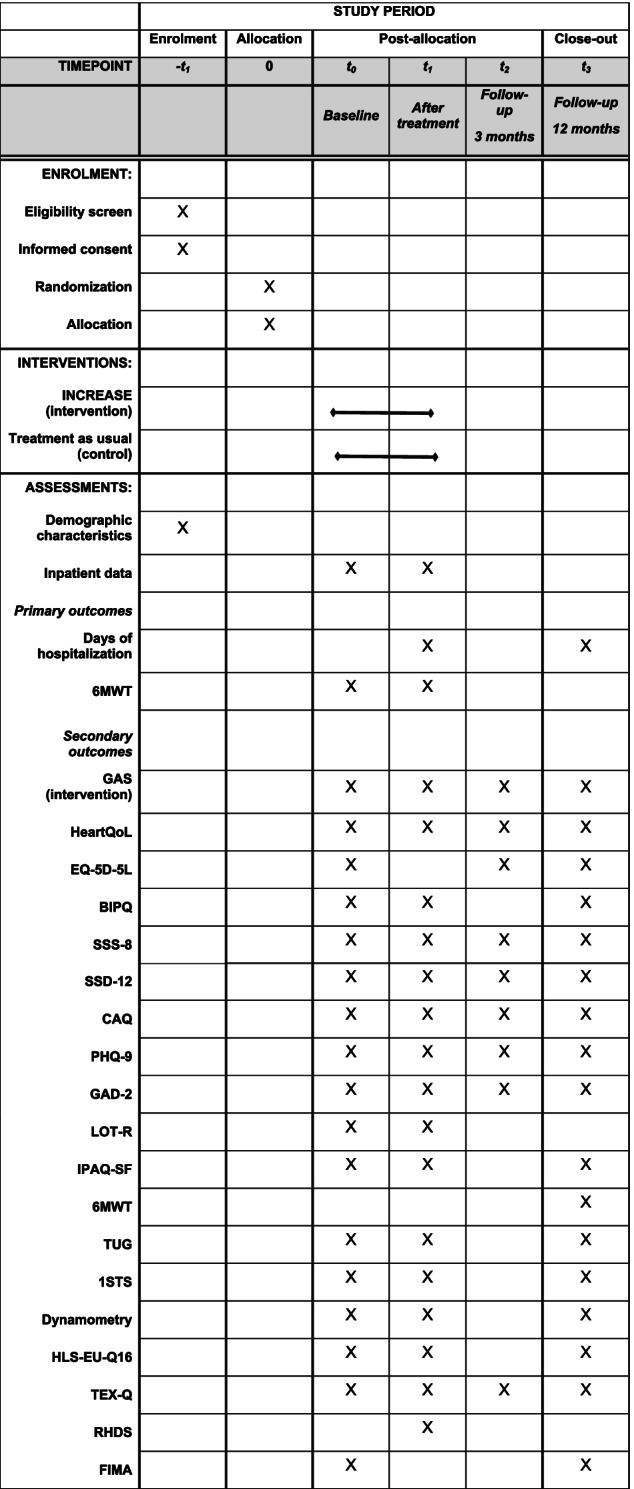
Schedule of enrolment, interventions, and assessments

*1STS* One Minute Sit to Stand Test, *6MWT* Six Minute Walk Test, *BIPQ* Brief Illness Perception Questionnaire, *CAQ* Cardiac Anxiety Questionnaire, *EQ-5D-5L* European Quality of Life 5 Dimensions 5 Level Version, *FIMA* Questionnaire for Health-Related Resource Use in an Elderly Population, *GAD-2* Generalized Anxiety Disorder-2, *GAS* Goal Attainment Scale, *HLS-EU-Q16* European Health Literacy Questionnaire, *IPAQ-SF* International Physical Activity Questionnaire Short Form, *LOT-R* Life Orientation Test –revised, *PHQ-9* Patient Health Questionnaire-9, *QoL* Quality of life, *RHDS* Readiness for Hospital Discharge, *SSD-12* Somatic Symptom Disorder – B Criteria Scale, *SSS-8* Somatic Symptom Scale-8, *TEX-Q* Treatment Expectation Questionnaire, *TUG* Timed Up and Go Test

### Data analysis

Data collection, processing, and storage will be according to valid regulations. Data will be entered by study nurses not involved in patient care and investigators ensuring principle of dual control must verify all data. An independent monitoring committee will review trial progress, will provide advice, and will supervise the trial including data integrity and safety. Statisticians not involved in the provision of care and blinded to the participants will perform analysis. Descriptives of the baseline and follow-up data will be presented in the whole study cohort as well as separately, according to the intervention and control groups. The type of presentation is defined by the scale of corresponding variable. For categorical variables, absolute and relative frequencies will be given. Median and interquartile range will be set for variables with ordinal scaling and a sufficient number of categories as well as for metric non-normally distributed variables. For metric normally distributed variables, the arithmetic mean and standard deviation will be presented. Descriptive *p*-values will be reported with respect to the baseline comparisons.

For both co-primary endpoints, two-factorial ANOVA models will be calculated, including the treatment group and study center as co-variates. From these models, the one-sided 97.5% confidence intervals for the mean differences will be calculated (for the hospital LOS assuming unequal variances, for the 6MWT assuming equal variances).

The absolute scale (i.e., mean total days in hospital in the intervention group minus mean total days in hospital in the control group) will be used for the first endpoint, while the relative scale ((mean 6MWT distance in the study group minus mean 6MWT distance in the control group) divided by the mean 6MWT distance in the control group) will be used for the definition of the 6MWT study co-endpoint. Thus, for the total days in hospital, negative values will indicate fewer days in hospital when receiving ERAS intervention; for the 6MWT walking distance, positive values will indicate longer walking distance in the ERAS intervention group. Accordingly, the null hypothesis regarding total days in hospital will be rejected when the upper limit of the associated confidence interval will be below zero. The null hypothesis regarding walking distance in the 6MWT will be rejected if the lower limit of the associated confidence interval will be above -0.15 (non-inferiority limit).

The intention-to-treat analysis will be performed. Additionally, the data will also be analyzed using a per protocol approach. No interim evaluations are scheduled.

## Discussion

This randomized controlled trial with a calculated sample size of 186 participants aims to examine the effectiveness of an ERAS approach in the cardiac surgery versus a standard of care treatment. The three key features in the specialized ERAS program are the following: a minimally invasive surgical access, an early extubation, and an intensive mobilization starting 3 h postoperatively. Primary outcome measures in the INCREASE trial will be (a) the total days in hospital due to cardiac causes during the first year after surgery and (b) the functional performance measured by the 6MWT.

So-called fast track programs in the cardiac surgery were introduced about two decades ago [48]. However, only few programs focus on minimally invasive surgery and in particular on the modern minimally invasive heart valve surgery [49]. Minimally invasive approaches in heart valve surgery have been demonstrated to be safe and associated with positive health outcomes [50]. Zaouter et al. evaluated their fast-track surgery pathway in a mini-invasive aortic valve replacement surgery using a non-randomized before-after-design. The post-implementation procedure which included a preoperative counseling and first mobilization on the day of surgery resulted in a significant shorter LOS as compared to a historical pre-implementation procedure using a minimally invasive surgical access but the first mobilization on the third postoperative day (i.e., LOS in the pre-implementation group was 10 days [9–13.5] vs. 7 days [6.5–8] in the post-implementation group, *p* < 0.001) [13].

In the present study, ERAS approach in the minimally invasive heart valve surgery will be evaluated using a randomized controlled approach. The results of this trial may help to reduce the high health-related burden and the treatment costs of heart valve diseases [51]. Besides the benefit of the minimally invasive access route, the INCREASE approach aims to follow an interprofessional care approach by bringing together the different health care professions. First, the INCREASE nurse as a coordinator of the whole treatment process and the primary contact person for the patients and their families connects the different stakeholders and plays a crucial role in the implementation of ERAS programs [52]. Second, the physiotherapeutic concept is an evidence-based approach with a focus on the daily physical activity throughout the whole treatment process. Early high-intensive mobilization can be safely and effectively adopted in the clinical routine [53, 54]. Current evidence suggests that an early mobilization is the critical component to fasten the postoperative recovery, to retain the functional capacity, and to prevent complications, while an additional respiratory therapy alone does not make any significant difference [55–57]. Third, the incorporation of a psychologist into the ERAS program is a new component in the INCREASE trial, which has not yet been reported. Of note, a previous study including patients who underwent coronary artery bypass grafting and received a pre-operative psychosomatic intervention demonstrated a significant improvement in the disability score [9] and markedly reduced LOS [8]. Such positive effects might be also supposed in patients undergoing heart valve surgery, which justifies the involvement of psychologists in the INCREASE study. Finally, using an individualized goal setting to drive the postoperative in-hospital stay and to educate the patient to acquire an active role in the ERAS program might lead to improved outcomes [58].

Despite these advantages, our study might have some limitations. The INCREASE pathway requires a PACU to guide the postoperative care process. Implementation of PACU instead of conventional intensive care unit has been shown to be beneficial in in the conceptualization of fast-track program [59]. Only few heart centers have a specialized PACU, which might limit the implementation in other institutions. Nonetheless, this study has a potential to provide a robust evidence for the usefulness of a PACU and its key role in the ERAS programs. Another possible limitation might be the definition of crucial components for the effectiveness of the INCREASE approach: The INCREASE is a multimodal process-oriented concept, which contains a myriad of specific components. Hence, attributing the different level of effects to one of the components in detail will be rather difficult.

One of the crucial work packages is the development of a clinical implementation guide for the INCREASE adoption in other heart centers. This might help overcoming some of the above-mentioned limitations in the adoption process. Clinical implementation will add on the clinical impact of the INCREASE study by providing practical implementation tips together with the evidence of the cumulative effect of the ERAS approach.

### Trial status

Recruitment started in July 2021 at the University Medical Centre Hamburg-Eppendorf and in November 2021 at the University Hospital Augsburg and is anticipated to continue until end of 2022. Follow-up will be finished by the end of 2023.

## Data Availability

The results of the study will be shared via publications and presentations taking into account the Recommendations for the Conduct, Reporting, Editing, and Publication of Scholarly work in Medical Journals. The data and additional material generated during the study will be available from the corresponding author on reasonable request.

## Abbreviations

1STS: One Minute Sit to Stand Test
6MWT: Six-Minute Walk Test
BIPQ: Brief Illness Perception Questionnaire
CAQ: Cardiac Anxiety Questionnaire
EQ-5D-5L: European Quality of Life 5 Dimensions 5 Level Version
ERAS: Enhanced recovery after surgery
FIMA: Fragebogen zur Inanspruchname medizinischer und nicht-medizinischer Versorgungsleistungen im Alter
GAD-2: Generalized Anxiety Disorder Scale, 2-item version
GAS: Goal Attainment Scale
HLS-EU-Q16: European Health Literacy Questionnaire
ICU: Intensive care unit
IPAQ-SF: International Physical Activity Questionnaire Short Form
LOS: Length of stay
LOT-R: Life Orientation Test—revised
LUCAS: Longitudinal Urban Cohort Ageing Study
PACU: Postoperative anesthesia care unit
PHQ-9: Patient Health Questionnaire -9
PONV: Postoperative nausea and vomiting
RHDS: Readiness for Hospital Discharge Scale
SD: Standard deviation
SSD-12: Somatic Symptom Disorder—B Criteria Scale
SSS-8: Somatic Symptom Scale—8
TAU: Treatment as usual
TEX-Q: Treatment Expectation Questionnaire
TUG: Timed Up and Go
