# Correction: Interdisciplinary and cross-sectoral perioperative care model in cardiac surgery: implementation in the setting of minimally invasive heart valve surgery (INCREASE)—study protocol for a randomized controlled trial

**DOI:** 10.1186/s13063-022-06813-9

**Published:** 2022-10-05

**Authors:** Susanne G. R. Klotz, Gesche Ketels, Christian A. Behrendt, Hans-Helmut Konig, Sebastian Kohlmann, Bernd Lowe, Johannes Petersen, Sina Stock, Eik Vettorazzi, Antonia Zapf, Inke Zastrow, Christian Zollner, Hermann Reichenspurner, Evaldas Girdauskas

**Affiliations:** 1 Department of Physiotherapy, University Medical Center Hamburg-Eppendorf, Martinistr. 52, 20246 Hamburg, Germany; 2 Research Group GermanVasc, Department of Vascular Medicine, University Heart and Vascular Center, University Medical Center Hamburg-Eppendorf, Martinistr. 52, 20246 Hamburg, Germany; 3 Department of Health Economics and Health Services Research, University Medical Center Hamburg-Eppendorf, Martinistr. 52, 20246 Hamburg, Germany; 4 Department of Psychosomatic Medicine and Psychotherapy, University Medical Center Hamburg-Eppendorf, Martinistr. 52, 20246 Hamburg, Germany; 5 Department of Cardiovascular Surgery, University Heart and Vascular Center, University Medical Center Hamburg-Eppendorf, Martinistr. 52, 20246 Hamburg, Germany; 6 Department of Cardiothoracic Surgery, University Hospital Augsburg, Stenglinstr. 2, 86156 Augsburg, Germany; 7 Department of Medical Biometry and Epidemiology, University Medical Center Hamburg-Eppendorf, Martinistr. 52, 20246 Hamburg, Germany; 8 Department of Patient and Care Management, University Medical Center Hamburg-Eppendorf, Martinistr. 52, 20246 Hamburg, Germany; 9 Department of Anesthesiology, Center of Anesthesiology and Intensive Care Medicine, University Medical Center Hamburg-Eppendorf, Martinistr. 52, 20246 Hamburg, Germany

**Correction: Trials 23, 528 (2022)**

**https://doi.org/10.1186/s13063-022-06455-x**

Following the publication of the original article [1], we were notified of a correction in the Preoperative components section (page 4, first column):

Original publication:

“Additionally, the diary comprises cognitive exercises for the period between the education session and the operation to support a cognitive-affective level, to develop individual coping strategies, and to become an active part in the treatment process.”

Revised version:

“Additionally, the INCREASE diary comprises expectation management strategies for the period between the education session and the operation to develop positive and realistic post-surgery expectations, to elaborate individual coping strategies, and to become an active part in the treatment process. This psychological intervention is mainly based on the EXPECT manual from the PSY-HEART trial (Salzmann et al. 2018, Laferton et al. 2013)”

Additionally, Fig. 1 was missing the labels for the time points of measurements (t0, t1, t2, and t3 instead of simply t). These have now been added.

The original article has been corrected.

## References

[1] Klotz (2022). Interdisciplinary and cross-sectoral perioperative care model in cardiac surgery: implementation in the setting of minimally invasive heart valve surgery (INCREASE)—study protocol for a randomized controlled trial. Trials..

